# Targeting myeloid-derived suppressor cells in combination with tumor cell vaccination predicts anti-tumor immunity and breast cancer dormancy: an in silico experiment

**DOI:** 10.1038/s41598-023-32554-z

**Published:** 2023-04-11

**Authors:** Reza Mehdizadeh, Seyed Peyman Shariatpanahi, Bahram Goliaei, Curzio Rüegg

**Affiliations:** 1 Institute of Biochemistry and Biophysics, University of Tehran, Tehran, Iran; 2 Laboratory of Experimental and Translational Oncology, Pathology, Department of Oncology, Microbiology and Immunology, Faculty of Sciences and Medicine, University of Fribourg, Fribourg, Switzerland

**Keywords:** Immunotherapy, Tumour immunology, Vaccines, Cell vaccines, Cancer immunotherapy, Programming language, Computational models, Breast cancer, Cancer microenvironment, Cancer models

## Abstract

Among the different breast cancer subsets, triple-negative breast cancer (TNBC) has the worst prognosis and limited options for targeted therapies. Immunotherapies are emerging as novel treatment opportunities for TNBC. However, the surging immune response elicited by immunotherapies to eradicate cancer cells can select resistant cancer cells, which may result in immune escape and tumor evolution and progression. Alternatively, maintaining the equilibrium phase of the immune response may be advantageous for keeping a long-term immune response in the presence of a small-size residual tumor. Myeloid-derived suppressor cells (MDSCs) are activated, expanded, and recruited to the tumor microenvironment by tumor-derived signals and can shape a pro-tumorigenic micro-environment by suppressing the innate and adaptive anti-tumor immune responses. We recently proposed a model describing immune-mediated breast cancer dormancy instigated by a vaccine consisting of dormant, immunogenic breast cancer cells derived from the murine 4T1 TNBC-like cell line. Strikingly, these 4T1-derived dormant cells recruited fewer MDSCs compared to aggressive 4T1 cells. Recent experimental studies demonstrated that inactivating MDSCs has a profound impact on reconstituting immune surveillance against the tumor. Here, we developed a deterministic mathematical model for simulating MDSCs depletion from mice bearing aggressive 4T1 tumors resulting in immunomodulation. Our computational simulations indicate that a vaccination strategy with a small number of tumor cells in combination with MDSC depletion can elicit an effective immune response suppressing the growth of a subsequent challenge with aggressive tumor cells, resulting in sustained tumor dormancy. The results predict a novel therapeutic opportunity based on the induction of effective anti-tumor immunity and tumor dormancy.

## Introduction

According to WHO, breast cancer is the most prevalent cancer in women worldwide. Among the different subtypes, triple-negative breast cancer (TNBC) is the most aggressive one with the worst clinical outcome^1^. In the absence of a validated targeted therapy, chemotherapy is still the standard of care for TNBC, and the development of drug resistance is a major problem resulting in cancer progression and eventual patient death. In recent years, researchers focused on overcoming resistance by exploring different combinations of multiple cytotoxic, targeted, and immune-stimulating therapies^2–4^.

The ability of the immune system to eliminate tumor cells has been emphasized since the 1950s^5^. The evolutionary dynamics of the immune response to cancer cells may eradicate cancer in the elimination phase, or cancer cells may evade the immunosurveillance phase, eventually resulting in a growing tumor and the formation of metastases^6^. Alternatively, immunoediting may also result in immunological dormancy, in which cancer cells are maintained as a small population under immunological control^7^. Importantly, it has been shown that the presence of tumor-infiltrating lymphocytes (TILs) in breast cancer, particularly in TNBC, correlates with a more favorable clinical outcome^8–10^.

Over the past decade, we have learned how the immune system can be exploited as a therapeutic tool in patients^11^. Immuno-oncology has been extensively considered and applied to treat advanced and metastatic tumors by stimulating the anti-tumor immune response with clinical benefits. Immunotherapies may directly target tumor cells.  Otherwise, cytokines and checkpoint inhibitors are used to boost the natural, innate anti-tumor immune response^12^. Cancer vaccines, as another option, can be generated from killing or modifying cancer cells removed during surgery in the lab and used as personalized vaccines to strengthen the immune response to control cancer growth, eliminate residual tumor cells, or convey long-term immune memory^13,14^. Immunotherapy is now emerging as an important component in the therapeutic management of TNBC^15^.

While the specific immune response to the tumor can inhibit cancer growth, the tumor-induced non-specific inflammation can promote tumor cell proliferation, invasion, and metastasis^16^. Neoplastic cells not only directly take advantage of non-specific inflammatory responses to the tumor resulting in enhanced growth and invasion^17^, but also actively exploit inflammatory cells, such as myeloid-derived suppressor cells (MDSCs), to suppress specific anti-tumor immune responses^18^. MDSCs are a heterogeneous group of immature bone marrow-derived cells of the myeloid lineage, which are expanded and released during pathological conditions, such as cancers, infections, and inflammatory diseases^19^. Studies have shown that breast tumor cells shape MDSCs in the bone marrow and increase their expansion and activation in the tumor microenvironment (TME) by releasing cytokines, such as G-CSF, IL-6, and TGF-β^18^. MDSCs suppress the activity of cytotoxic T cells and NK cells in the TME through various mechanisms, including direct cell contact and recruitment of regulatory T cells (Treg). MDSCs also play a role in promoting angiogenesis, tumor invasion, and metastasis^20,21^. MDSC infiltration in the TME and subsequent suppression of the anti-tumor immune response are consistently associated with a poor clinical prognosis^22^.

An experimental study by Lan et al.^23^ has shown that a murine quiescent breast cancer cell line, MR20, derived from the parental 4T1 line, a preclinical model of TNBC, recruits fewer MDSCs to the TME than the 4T1 parental line itself. The lack of MDSC mobilization in the MR20 model is a critical factor permitting the development of an effective anti-tumor immune response resulting in sustained cancer dormancy^23^. The 4T1 model is exceptional and unique in the magnitude of MDSCs mobilization, and MDSCs’ role in 4T1 tumor progression is well documented^24–30^. Srivastava et al. showed that MDSC depletion has extensive impacts on strengthening the immune response, inhibiting angiogenesis, and suppressing LLC tumor growth and metastasis. They also found a positive effect of MDSC depletion on a therapeutic cancer vaccination outcome^31^. Further, Ravindranathan et al. confirmed that impairing an MDSC-stimulating and -accumulating factor in 4T1 cells increases immunogenicity and leads to a protective anti-tumor effect^32^.

We recently presented a mathematical model in which we proposed a vaccination approach using in vitro chemotherapy-treated cancer cells that theoretically and experimentally recruited fewer MDSCs in TME and were dormant in vivo. The vaccine enhanced the anti-tumor immune response and consequently repressed tumor growth^33^. Mathematical models and in silico studies are advantageous methods to shed light on the complex interplay between tumor cells and immune infiltrates and to present future therapeutic interventions. Although there are growing theoretical efforts to consider the role of MDSC in the immunosuppressive TME and its impact on the failure of immunotherapies, modeling of targeting MDSC as a potential treatment is still limited^34–37^. Here, we used ordinary differential equations (ODEs) to mathematically model the effect of MDSC depletion on tumor growth inhibition and to evaluate the synergy of preconditioning with aggressive cancer cells and simultaneous MDSC elimination on long-term cancer dormancy. These results show the potency of MDSC depletion, combined with a low-dose cancer cells injection, to strengthen the anti-tumor immune response and evoke sustained immune-mediated tumor dormancy.

## Methods

### Previously published experimental and modeling results

When Lan et al.^23^ orthotopically injected 5 × 10^4^ aggressive 4T1 cells into BALB/c mice, the immune profiling of tumor-bearing mice indicated an immunosuppressive environment enriched in MDSCs. In contrast, the immune profiling of tumor-free mice inoculated with an in vitro chemotherapy-treated cell line (MR20) derived from 4T1 cells indicated anti-tumor immune cell infiltration resulting in a state of immunological dormancy. It was proposed that type I interferon (IFN-I) response may suppress MDSCs recruitment and switch the immune response toward cytotoxicity^23,38^.

Based on the above experimental observations, a mathematical model that describe the tumor growth and immunological dormancy states was generated^33^. The model also proposed prophylactic and therapeutic tumor cell-based vaccine schedules resulting in the increased number and anti-tumor activity of T-cells.

### Assumptions

This study aims to make a mathematical model of MDSC depletion from the TME and its effect on the anti-tumor immune response. This model is based on the experimental results by Lan et al.^23^ and the theoretical models by Mehdizadeh et al.^33^ and Shariatpanahi et al.^34^. It aims at generating novel putative therapeutic opportunities. For developing the model, the following assumptions were considered:

1. The proliferation rate minus the natural death rate is represented by the net tumor growth rate.
2. MDSC recruitment, expansion, and activation in the tumor bed are induced by tumor cells.
3. The immune cells represent the directly cytotoxic cells: NK cells and CD8^+^ T lymphocytes (CTL).
4. Immune cells can be inactivated by tumor cells, mainly via MDSCs’ activity.

The choice of including MDSC, NK cells, and T cells, while excluding other cells of the TME, such as monocytes, dendritic cells, fibroblasts, or endothelial cells, was dictated by the fact that experimental data were only available for the formers.

### Mathematical model

To construct the model, a system of three ODEs, which express the change of cell populations, was used. The three variables of the model were: cancer cells (C), MDSCs (M), and effector immune cells (I). The evolution of tumor cells in the TME is represented by Eq. (1).

1 $$\frac{dC}{{dt}} = aC\ln (\frac{Cmax}{C}) - \tau IC,$$

A Gompertz growth term, $aC \ln (\frac{Cmax}{C})$, points out the growth of the cancer population, in which $a$ is the net tumor growth rate and $Cmax$ is the maximum tumor size. The term takes into account the increase in tumor size over time, as well as the natural slowing of growth that occurs as the tumor matures according to insufficient nutrient supply and cell-contact inhibition. The Gompertzian growth model is typically used in mathematical models of breast cancer and fits best with the published experimental results^39,40^. The mass action term, $- \tau IC$, indicates the killing potency of cancer cells by immune cells, and $\tau$ is the rate.

MDSCs are defined separately from immune cells as an independent variable, $M$, and their dynamics in the TME over time is investigated using a separate equation.

2 $$\frac{dM}{{dt}} = \alpha_{m} C - \beta M,$$

In the above equation, the first term shows that the presence of MDSCs in the TME depends on the presence of tumor cells by the $\alpha_{m}$ ratio. Then, the second term states the natural death of MDSCs at the rate of $\beta$.

To simplify and reduce the number of uncertain parameters, the population dynamics of immune effector cells, i.e., NK cells and T lymphocytes, is expressed by a single equation.

3 $$\frac{dI}{{dt}} = s + I\left( {q\left( {\frac{{C^{2} }}{{l + C^{2} }}} \right)} \right){ } - {\text{nI}} - \mu MI,$$

S is a constant source of immune cells in the TME. Inducing the immune cells’ recruitment to the TME by the cancer cells is described by a saturation kinetic, $I\left( {q\left( {\frac{{C^{2} }}{{l + C^{2} }}} \right)} \right)$, in which $q$ and $l$ are the maximum recruitment rate and the steepness coefficients, respectively. The saturation term is modeled as a Michaelis–Menten kinetic, which relates the presence of effector immune cells to the concentration of tumor cells. It represents a sigmoid function, from where the immune system’s response is low until the number of tumor cells reaches a threshold, after which the response rapidly increases until it reaches a maximum value. The saturation term helps to describe the diminishing returns of the immune system when it is overwhelmed by the tumor cells. The term $\it - {{nI}}$ represents the natural decay of immune cells at the rate of $n$, and the term $- \mu MI$ is a mass action description of immune cells inactivation by MDSCs activity and by a rate of $\mu$.

### Initial conditions and parameters

The parameters were obtained from the literature and estimated by fitting them to the experimental data from Ref.^23^. To replicate the experimental condition of Lan et al.’s study^23^ and obtain more accurate parameters, the initial number of 4T1 tumor cells was set to 5 × 10^4^, and the initial number of cytotoxic immune cells and MDSCs was estimated at 7.7 × 10^5^ and 6.4 × 10^4^ cells, respectively.

Fitting by the least-square distance was performed during an estimation process using optimization algorithms in MATLAB. All the parameters, their values, descriptions, and references are presented in Table 1.

**Table 1:** Parameter values.

Parameters	Units	Estimated value	Description	Source
$a$	Day^−1^	0.38 × 10^–1^	(4T1) tumor growth rate	^33^
$C_{\max }$	Cell	10^9^	Maximum tumor size	^33^
$\tau$	Cell^−1^ Day^−1^	0.16 × 10^–7^	Tumor cells’ kill rate by immune cells	Estimated from ^33^
$\alpha_{m}$	Day^−1^	2.2 × 10^–1^	MDSC recruitment rate by proliferative tumor cells	Estimated
$\beta$	Day^−1^	0.35	Death rate of MDSCs	Estimated from ^34^
$s$	Cell day^−1^	1.3 × 10^4^	Constant source of immune cells	^33,34,41,42^
$q$	Day^−1^	12 × 10^–2^	Maximum immune effector cells recruitment rate by tumor cells	Estimated from ^33^
$l$	Cell^2^	2.02 × 10^7^	Steepness coefficient of the immune effector cell recruitment curve	^33,34,41,42^
$n$	Day^−1^	4.12 × 10^–2^	Death rate of immune effector cells	^33,34,41,42^
$\mu$	Cell^−1^ Day^−1^	4.5 × 10^–8^	Immune effector cell inactivation rate by MDSCs	Estimated

### Simulations

To demonstrate the reliability of the model, the following series of computational simulations were performed by the Runge–Kutta methods and by the ode23s solver in MATLAB R2018b software.

In the first series of simulations, the tumor growth in the presence and absence (removal) of MDSCs was simulated by simultaneously solving all the equations. The potential power of MDSC depletion in inducing an immune response, halting tumor growth, and producing subsequent tumor dormancy was investigated by the second series of computational experiments. Finally, the third series of simulations addressed the synergistically preconditioning effect of tumor cell injection plus MDSC depletion on the subsequent invasive tumor growth.

## Results

### Simulations of proliferative tumor growth in the presence and absence of MDSCs

**Figure 1:**
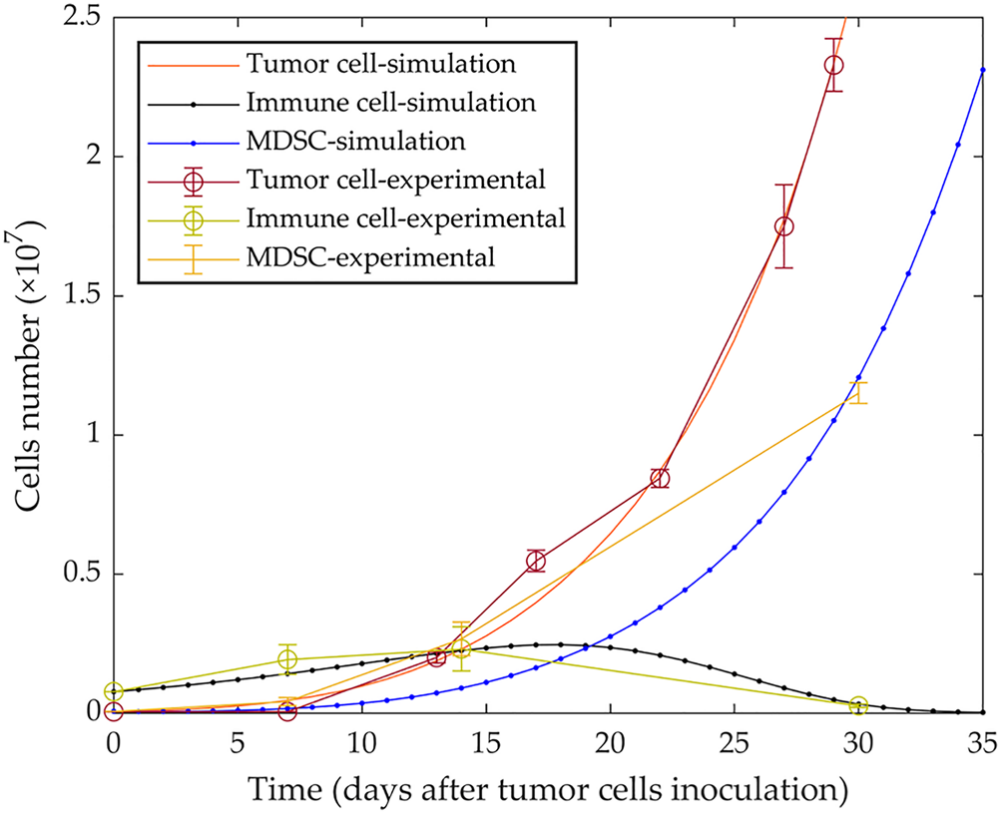
Mathematical simulation of tumor growth in the presence of MDSCs. By simulating the injection of 5 × 10^4^ 4T1 tumor cells in immune-competent mice, tumor growth dynamics, immune response, and MDSCs are shown. The published experimental data^23^ are represented as mean ± SEM. Myeloid-derived suppressor cell: MDSC.

Due to the importance of MDSC cells, present in the TME, on the tumor growth dynamics and the suppression of the anti-tumor immune response was examined by solving the model's equations. The first simulation replicates the experimental data and shows that cancer cells can escape immune surveillance and grow in the presence of MDSCs (Fig. 1). The experimental data^23^ were used to calibrate the parameters.

**Figure 2:**
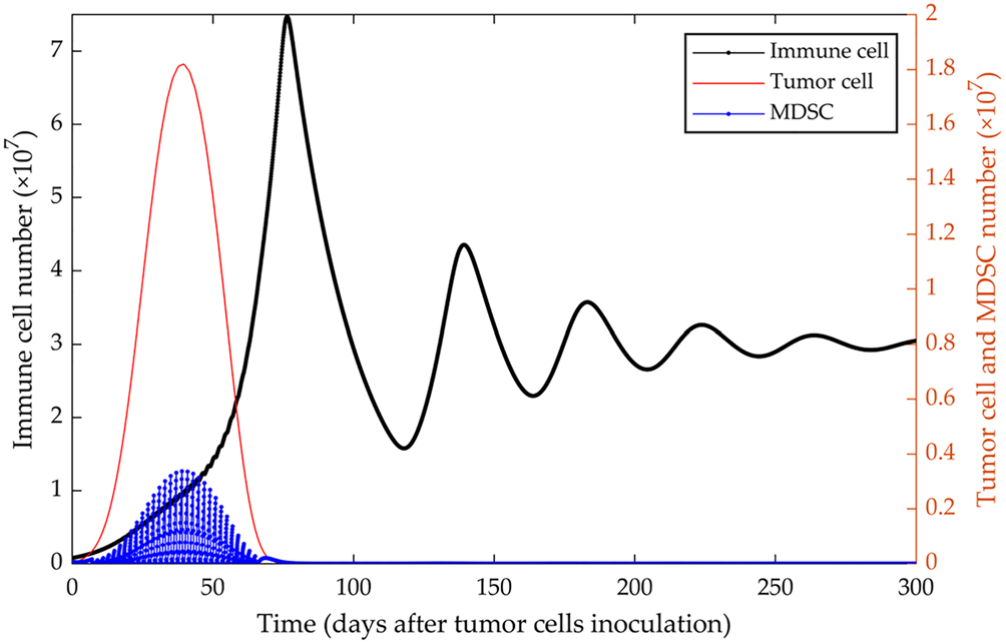
Simulation of MDSC depletion from tumor-bearing mice. By simulating the depletion of MDSCs every 48 h and for 2 months starting from day 7 after the injection of 5 × 10^4^ 4T1 tumor cells in immunocompetent mice, the tumor growth is halted. The left y-axis represents the number of immune cells, and the right y-axis shows the number of MDSCs and tumor cells.

On the other hand, simulation of MDSCs depletion every 48 h, starting 1 week after inoculation of 5 × 10^4^ proliferative 4T1 tumor cells, results in increasing anti-tumor immune activity and eventually tumor growth control (Fig. 2), in agreement with the proven effect of MDSC cells in suppressing anti-tumor immune response in the microenvironment, and in promoting the growth of 4T1 tumors as reported by Lan et al.^23^ and others^30^.

As these results suggested that MDSC depletion may be a potentially valuable therapeutic intervention, we set up other simulations to test how we could further optimize this approach.

### Simulations of the potency of MDSC depletion for therapeutic intent

**Figure 3:**
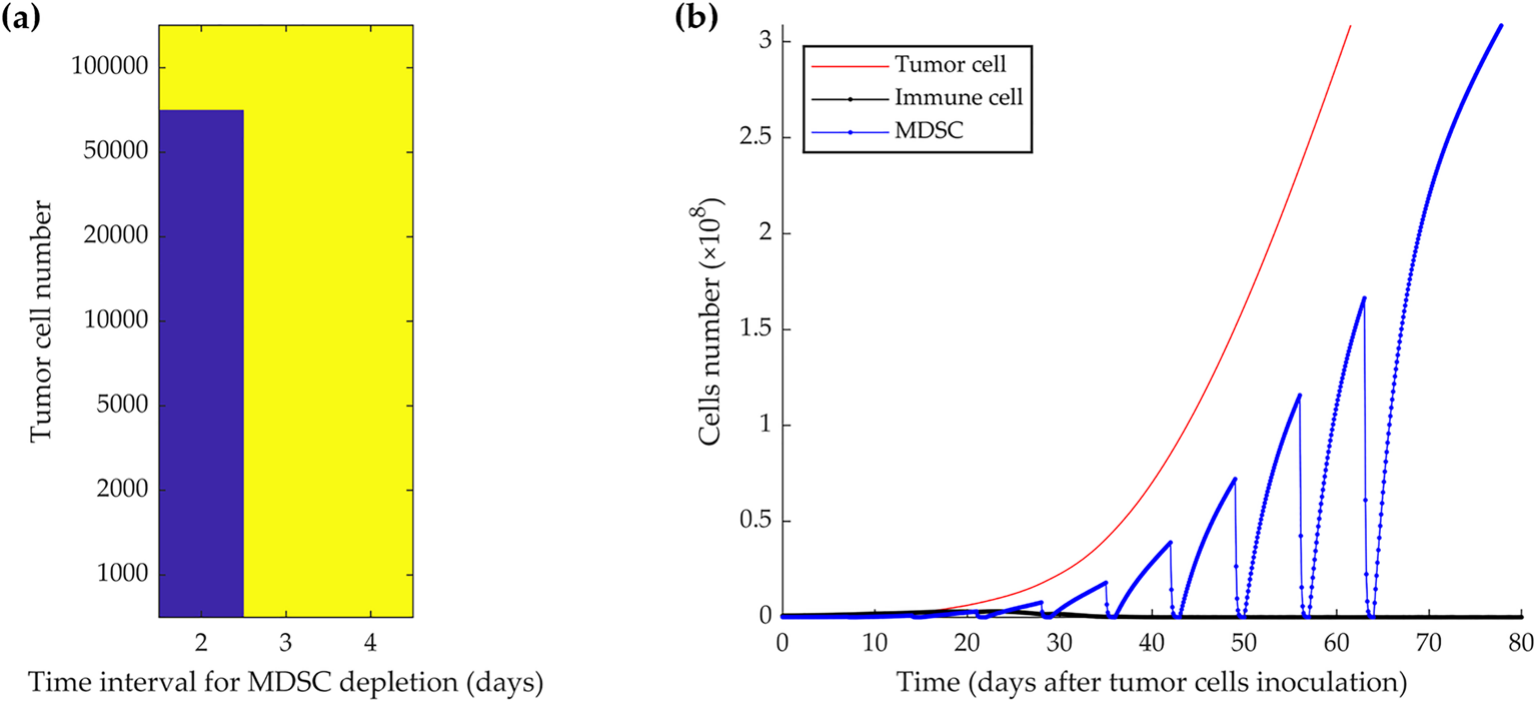
Simulation of how different time intervals of MDSC depletion from mice bearing growing tumors can induce cancer dormancy or fail to control tumor growth. For up to 70,000 initial tumor cells and starting depletion from day 7, every 2 days depletion during 2 months resulted in the induction of permanent dormancy, while depletion every 3 or 4 days did not (**a**). Color-coded events: blue, tumor dormancy; yellow, tumor growth. The time course simulation experiment shows that 3 days intervals in the depletion schedule, starting at day seven, result in tumor growth and immunosuppression after 5 × 10^4^ tumor cells injection (**b**).

By analyzing the frequency of depletion, it emerged that for up to 70,000 initially injected tumor cells, the tumor growth stops when MDSC depletion is done every 2 days, starting from different time points (Fig. 3a). The left panel of the figure shows the dormancy state in blue and the escape state in yellow. As a demonstration of the importance of the 2 days interval, Fig. 3b depicts a scenario in which MDSCs are depleted every 3 days, starting from day 7 after 5 × 10^4^ tumor cells injection. This depletion schedule failed to control tumor growth.

**Figure 4:**
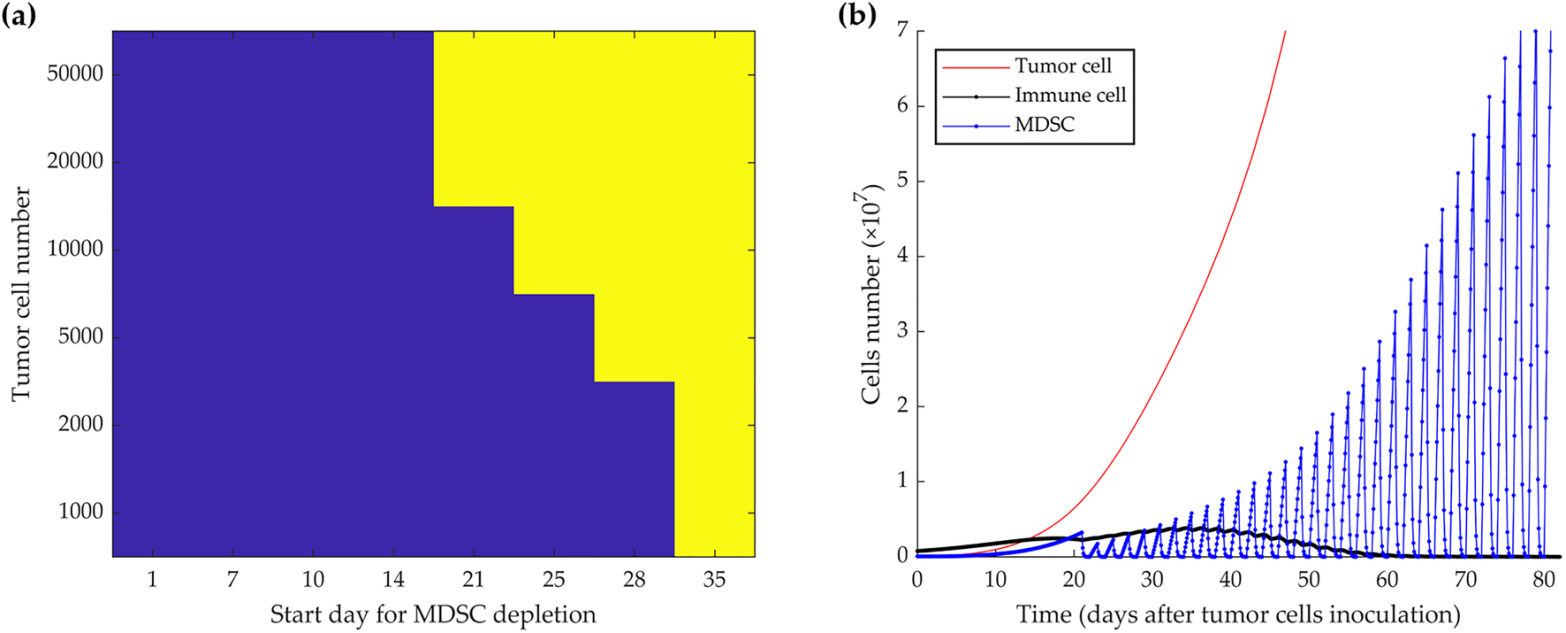
Simulation of MDSC-depletion from tumor-bearing mice every 2 days for 2 months with different starting days after tumor cell injection. Different start points impact cell fates according to the different initial tumor sizes (**a**). Color-coded events: blue, tumor dormancy; yellow, tumor growth. MDSC depletion from day 21 after 5 × 10^4^ tumor cells inoculation could not control the tumor growth (**b**).

To identify the most effective starting point for MDSC depletion to control tumor growth, we modeled the tumor fate based on the different initial tumor sizes (i.e., number of injected cancer cells) and different starting days of the depletion, then repeated every 48 h for 2 months. According to the initial tumor size, the depletion schedule can start up to nearly 30 days after the initial cancer cells’ implantation if the number of tumor cells is sufficiently low (Fig. 4a). The result indicates that MDSC depletion, from up to 2 weeks after injection of 5 × 10^4^ proliferating tumor cells, still induces dormancy, while a start at later time points (i.e., 3 weeks) results in progressive tumor growth (Fig. 4a, b).

**Figure 5:**
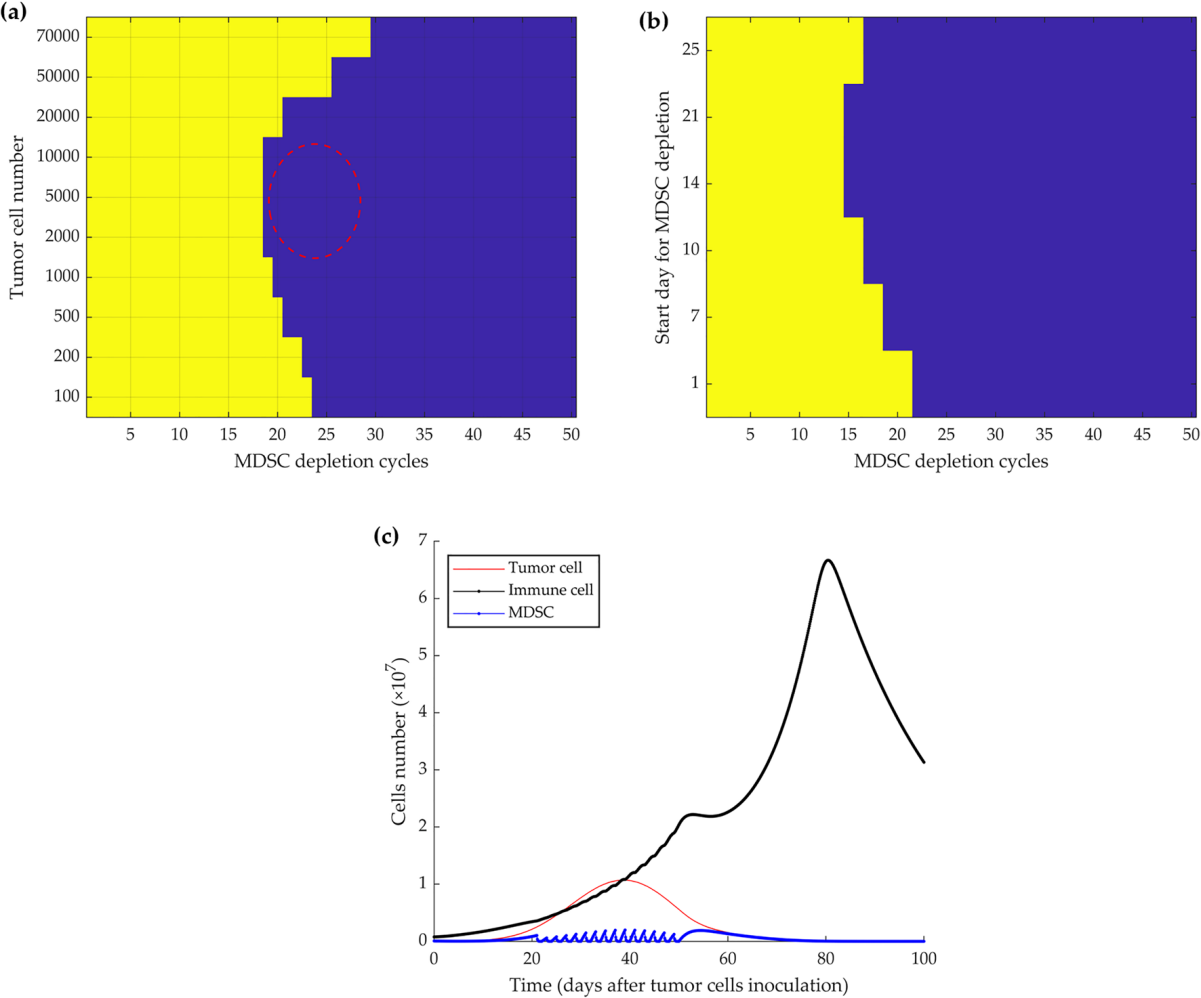
Simulation of an effective schedule of MDSC-depletion in mice bearing proliferative tumor cells. The necessary repetition of depletion every 2 days starting from day 7, is shown based on different initial tumor sizes (**a**) and the depletion starting point. Mice were injected with 5000 tumor cells (**b**). Color-coded events: blue, dormancy; yellow, tumor growth. Depletion of MDSC every 48 h from day 21 for 15 cycles in mice initially injected with 5000 tumor cells results in tumor growth control and permanent dormancy (**c**).

Figure 5a shows that the optimum initial size to induce immunological dormancy with minimum necessary depletion cycles from day 7 is between 2000 and 10,000 cells, the red dotted circle. As the lower number of cells induces a lower immune response, depleting MDSC in the early days after tumor injection has no significant impact on the tumor growth dynamics. Thus, it needs more depletion cycles to reach the critical time points for inducing tumor dormancy. By injecting 5000 tumor cells, minimum repetitions for depletion, 15 times, occurred by starting the depletion schedule during the 3rd week after tumor inoculation (Fig. 5b). In Fig. 5c, the immune-induced dormancy and tumor control are achieved by every 48 h of MDSC cell removal from day 21 for 1-month.

### Simulations of the preconditioning effect of proliferative tumor cells combined with MDSC depletion

**Figure 6:**
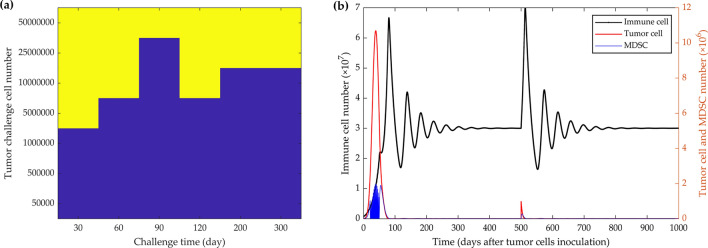
Simulation of the preconditioning effect of proliferative tumor cells in combination with MDSC-depletion on the tumor challenge fate. According to the challenge size and time, the stimulation of an immune response, with 5 × 10^3^ tumor cells inoculation and depletion of MDSCs every 48 h from day 21 for 1 month, prevent tumor cell challenges from generating tumors for several years (**a**). Color-coded events: blue, dormancy; yellow, tumor growth. Preconditioning with 5 × 10^3^ tumor cell inoculation and depletion of MDSCs every 48 h from day 21 for 1-month duration resulted in long-term immunity and growth control of 1 million tumoral challenging cells injected 500 days after the initial (preconditioning) tumor cells injection (**b**). The left y-axis represents the number of immune cells, and the right y-axis the number of MDSCs (blue curve) and tumor cells (red curve).

To simulate how the schedule could prevent subsequent tumor growth, Fig. 6a demonstrates the tumor fate based on the tumor challenge size and the time in which the challenge occurred. MDSC depletion for 1 month after 5000 tumor cells injection can induce an immune response that prevents subsequent tumor cell challenges from generating growing tumors for up to 1000 days (Fig. 6b).

## Discussion

Breast cancer is a leading cause of cancer mortality in women worldwide^43^. While breast cancer subtypes expressing the estrogen and possibly progesterone receptors (ER/PR) or human epidermal growth factor receptor 2 (HER2) have curative options with targeted adjuvant therapies, the TNBC subtype has limited targeted therapies, and most patients are still mainly treated with chemotherapy^44^. Recurrence, treatment resistance, and development of metastases are the main causes of breast cancer-related death, particularly for TNBC^45^.

Immunotherapy may bring new hope for TNBC treatment. The rationale behind cancer immunotherapies is to unleash and strengthen the natural immune response against tumor cells to eliminate tumor cells or to induce immune-mediated dormancy and achieve long-term control of tumor growth and recurrence^11^. It is well-established that tumor-mobilized MDSCs, recruited to and activated within the primary and metastatic TME, play a pivotal role in suppressing the anti-tumor immune response, thereby facilitating tumor cells' growth, recurrence, and metastasis^46^. Accordingly, targeting MDSCs could potentially contribute to cancer control by inducing a strong anti-tumor immune activity^47,48^.

In this study, we used an ODE model to study the effect of MDSC depletion on tumor growth, anti-tumor immune response and sustained cancer dormancy. We also explored the potential synergy between preconditioning with aggressive cancer cells and the simultaneous depletion of MDSCs. To this end, we decided to generate a simplified model that considers T cells and NK cells totally as effector immune cells and MDSC cells, for which we had experimental data to build upon. Other cells of the TME that may also impact the immune response, including monocytes, dendritic cells, and endothelial cells, were not considered either because we had no experimental data or were irrelevant in the in vivo model^23^. With this simplified model, we mathematically analyzed the dynamic of immune effector cells and the suppressing effect of MDSCs on them using Eq. (3). It was assumed that the tumor's suppressor effect on immune effector cells only occurs through MDSC cells, while the inhibitory effect of MDSC on antigen-presenting cells (APC) was ignored. First, we used this mathematical model to reproduce the effect of MDSC cells on the dynamics of tumor growth and immune reaction, as reported by Lan et al.^23^ (Fig. 1).

By systematically removing MDSCs with intraperitoneal injecting antibodies directed against Gr1, a cell surface antigen expressed by mouse MDSC, every 48 h for 4 weeks from day 7 after orthotopic inoculation of 10,000 3LL lung cancer cells, Srivastava et al. showed that tumor growth extensively decreased and remained controlled, accompanied by a significant reduction of angiogenic signals and increased apoptotic activity. In parallel, the number and functionality of the immune effector cells and APCs presented in the TME improved^31^. Our model’s simulation of depleting MDSCs replicated this experimental outcome and thus demonstrated the adequacy of depleting MDSCs in controlling the aggressive 4T1 tumors (Fig. 2).

Countering or inhibiting MDSCs’ effects by systemic depletion of MDSCs, or selectively blocking their recruitment, expansion, and activation in the TME^48,49^ by repeated injection of antibodies is an invasive, costly, and time-consuming approach for mice and humans. Therefore, decreasing the frequency of cell depletion would be the first step toward the optimization of the process. The model simulation results demonstrated that MDSCs depletion, every 2 days, could control the growth of up to initially injected 70,000 tumor cells and induce sustained immune dormancy (Fig. 3). Depending on the initial tumor cell burden, MDSC depletion can be initiated up to around 30 days after the initial tumor cell injection (Fig. 4). According to Srivastava et al.'s study, the necessary repetition of MDSCs depletion starting 1 week after tumor inoculation was re-evaluated by modulating the initial number of injected tumor cells. Our results indicate the minimum required depletion cycles from day 7, i.e., 19 times, to instigate dormancy, is achieved when the initial number of tumor cells is low, between 2000 and 10,000 (Fig. 5a). An efficient starting point was determined to be the 3rd week after 5000 initial tumor cells injection and consisted of 15 removal cycles (Fig. 5b). Thus, by reducing the initial number of injected tumor cells and delaying the start of the MDSC depletion schedule, the number of necessary depletion cycles was decreased to a 1-month depletion schedule (Fig. 5c).

In another experiment, Ravindranathan et al.^32^ concluded that the increased tumor-derived factors, especially higher levels of granulocyte colony-stimulating factors (G-CSF), are the main reason for the highly splenic and tumor site accumulation of MDSCs and eventually the impairment of the immune response. By deleting G-CSF expression from 4T1 cells, they successfully controlled tumor growth, increased immunogenicity, and produced a vaccine-like protective effect on tumor cells^32^. To test the potency of the dormancy state in suppressing tumor growth at a later time point and preventing recurrence, we analyzed the effect of MDSCs depletion on tumor challenge according to the tumor challenge size and time after the first cancer cell injection and MDSCs depletion (Fig. 6a). The results demonstrate that MDSC depletion every other day, starting from day 21 for one month after 5000 initial tumor cells inoculation, can effectively generate an immune response to produce sustained dormancy and an immune protective effect to prevent tumor growth or relapses upon tumor rechallenges at different time points (Fig. 6a, b).

Srivastava et al. also compared the results of employing bone marrow adherent cells, which have been activated for a specific tumor antigen, as an immunogenic vaccine alone or in combination with MDSC depletion. By dual intervention strategy, the results have shown the synergetic effects, including an increased tumor growth inhibition rate resulting in the complete rejection of primary tumors in 50% of mice and the rejection of tumor rechallenges. They also demonstrated increased leukocytic infiltration and tumor staining of cleaved caspase 3, a signal for increased tumor cell apoptosis^31^. The simulation’s results of MDSC depletion in combination with an immunogenic tumor cell-based vaccine, as previously described in^33^, confirmed the synergistic effects of inducing a powerful immune response and growth inhibition of primary tumors and subsequent challenges. However, the results are not shown here due to the simplification of the presented model.

The presence of TILs and PD1/PDL1 expression in the tumor microenvironment of TNBC has been reported in many studies to correlate with a better outcome and to predict response to immunotherapy with anti-PD1/PDL1 inhibitors^50–56^. Initial studies of checkpoint inhibition monotherapy demonstrated limited response in patients with advanced PD-L1^+^ TNBC but failed to provide a survival advantage over chemotherapy. A combination of anti-PD1 with chemoimmunotherapy has been shown more successful and is now an approved therapy for PD-L1 positive advanced TNBC. Subsequently, anti-PD1 in combination with neoadjuvant chemotherapy was approved for high-risk early-stage TNBC for curative intent. In spite of these successes, responses to check point inhibitors remain limited to a subset of TNBC patients^57–59^. As TILs frequency and PD1/PDL1 expression are not sufficient to predict response efforts have been concentrated on identifying additional predictive markers. The cellular composition of the TME, the RNA gene expression profiling of T cells, aberrant chemokine and cytokine expression (i.e., CCL7, IL-33), and mutational tumor burden appear to play an important role in determining the efficacy of check point inhibitors-based immunotherapies^50,51,60^.

However, the strategy to maximally activate the immune system to increase the killing of cancer cells for full eradication may result in counterproductive outcomes due to the emergence of immune-resistant tumor cells escaping immune control^61^. Inducing and maintaining immune-mediated tumor dormancy may be considered a therapeutic alternative to aggressive cytotoxic- or immunotherapies^62,63^. Based on our results, we present a theoretical model of targeting MDSC in the presence of a low tumor burden to increase activated TILs and instigate a sustained immunity capable of controlling the growth of a large tumor mass at later time points. This approach could be translated into the clinic by suppressing MDSC activity after initial conventional treatments, e.g., surgery and chemotherapy, to increase the immune response against residual proliferative cells resulting in asymptomatic long-term immunological dormancy and reduced metastasis^64,65^. A monoclonal anti-CD33 antibody (gemtuzumab), against a surface marker of human myeloid cells, conjugated with a toxin (ozogamicin) has successfully depleted the CD33-expressing MDSCs and restored the capacity of T cell immunity and immunotherapy against cancers in clinical trials^66^.

## Conclusion

Immunotherapies based on diverse strategies have been designed, improved, and confirmed as therapeutic options for various solid and hematological cancers, in particular melanoma and lung tumors. The accumulation of MDSCs in the TME is considered a potential cause of cancer immunotherapy failure. Experimental studies demonstrated that MDSC differentiation, inactivation, or depletion results in a more effective immune response and improved tumor control. In order to improve the efficacy of immune-modulating therapies, it is essential to develop approaches to suppress MDSCs that can be translated into clinical trials. This paper presents a simplified theoretical model, which emphasizes the paramount immune-suppressing effect of tumor-mobilized and -activated MDSCs in suppressing the anti-tumor immune response. Additionally, it postulates that depletion of MDSCs in combination with a low-dose cancer cells burden produces a strong and prolonged anti-tumor immune response resulting in sustained immune-mediated dormancy. This simplified model does not consider all immunologically-relevant cells of the TME and may not be definitive. However, our in silico experiment and results provide insights for pursuing in vivo research to study MDSC suppressing in combination with other immune-inducing strategies, e.g., activating type I interferon response, vaccination, and immune checkpoint inhibition.

## Data Availability

Data are available from Reza Mehdizadeh on reasonable request.
